# Pediatric Stercoral Colitis and Acute Kidney Injury From Chronic Constipation

**DOI:** 10.1177/00099228241226501

**Published:** 2024-01-23

**Authors:** Daniel Kade Derrick, Leen Azeez, Myriam Barragan

**Affiliations:** 1 University of Texas Health Science Center at San Antonio, San Antonio, TX, USA

**Educational Objectives**

- Recognize conditions such as stercoral colitis that may be associated with the adult population can also occur in children with the correct risk factors or exposures.
- Review the diagnosis of chronic constipation and how it is widespread, underdiagnosed, and treatable and can lead to long term sequelae if not recognized.

## Case Description

A 15-year-old female with a past medical history of iron deficiency anemia and 3 years of constipation presents to the emergency department for abdominal pain of 1 week duration and difficulty urinating. She states for the last week she has been unable to empty her bladder and has not urinated for at least 24 hours. Her last bowel movement was today and was small and hard. The patient denies fever, chills, nausea, vomiting, and hematuria. Vitals are as follows: Temp 36.8°C (98.2°F), BP 90/60 mm Hg, HR 146 beats per minute, RR 18 breaths per minute, and SpO_2_ 97% on room air. On physical examination, the patient appears pale and thin, with dry mucous membranes. Her abdomen is distended without any skin changes with a large, nonballotable, firm, tender mass present in the right lower quadrant. No bruit is heard overlying the mass.

On presentation, the patient weighed 44.7 kg (14.49% percentile on the CDC Girls 2-20 Years Weight for Age chart), down from 46.2 kg (21.63% percentile) almost 6 weeks prior on a well child care visit.

**Figure 1.**
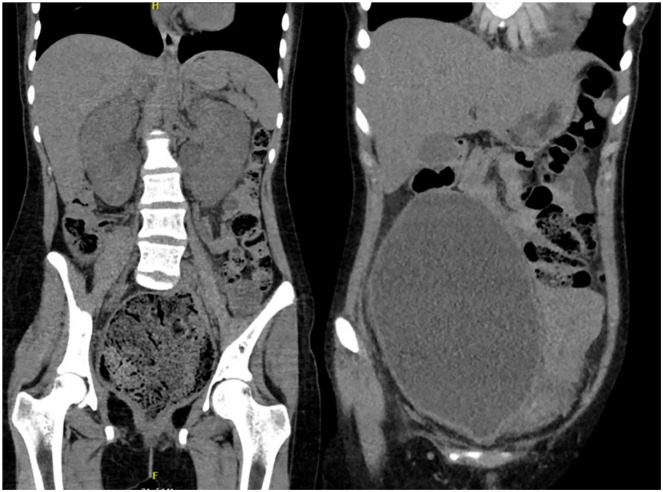
A coronal CT showing a rectal fecaloma (left) causing urethral obstruction and a massively dilated urinary bladder (right).

Lab studies are ordered, including complete blood count (CBC), electrolytes, TSH/T4, an iron panel, urinalysis, and culture, which are significant for sodium 128 mmol/L (low), a creatinine of 5.27 mg/dL (very high), and blood urea nitrogen (BUN) of 88 mg/dL (very high), albumin 2.6 g/dL (low), hemoglobin 8.5 g/dL (low), mean corpuscular volume of 69.8 fL (low), and 3+ urine protein. C-reactive protein is found to be 253.30 mg/L (high) with a sedimentation rate of 120 mm/hr (high). Abdominal computed tomography (CT) scan is performed and demonstrates a large rectal stool burden measuring 10 cm × 9 cm resulting in obstruction of the bladder with a very enlarged bladder (see Figure 1), along with mild bilateral hydronephrosis. Foley placement is attempted but unsuccessful by both nursing and urology. A 300-mL enema of cottonseed oil, docusate, and normal saline is administered, which facilitates passage of a small volume of stool and eventual foley catheter placement by urology with drainage of 4L of blood-tinged urine.

**Figure 2.**
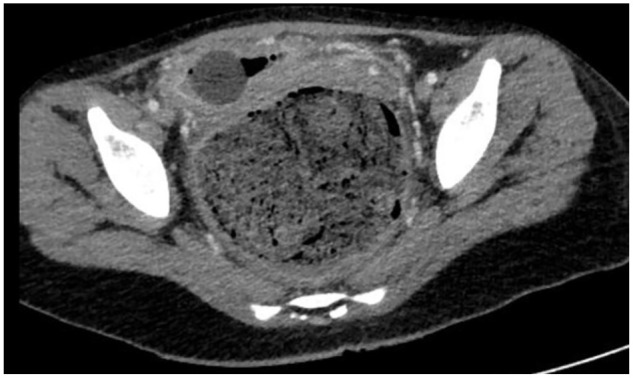
An axial CT showing an unchanged rectal fecaloma.

The patient is admitted to the pediatric team for continued management and the pediatric GI team is consulted for administration of a bowel cleanout with 2 more enemas, senna 17.2 mg orally twice a day, and oral polyethylene glycol 15 capfuls in 64 oz of fluids over 4 hours, which is poorly tolerated. Urine culture, from initial presentation, was found to be positive for *Escherichia coli* and *Enterococcus faecalis*, and antibiotic therapy with metronidazole and ciprofloxacin was thereby initiated, after empiric ceftriaxone treatment. After multiple days in the hospital and unsuccessful bowel cleanout demonstrated by CT showing a largely unchanged fecaloma of 9.5 cm × 8 cm (see Figure 2), the patient is taken to the OR for manual fecal disimpaction, colonoscopy, and esophagogastroduodenoscopy.

## Discussion

### Differential Diagnoses

Our patient first presented with hyponatremia, acute kidney injury (AKI), iron deficiency anemia, and hypoalbuminemia. A urine analysis from admission revealed a urinary tract infection with *E. coli* and *E. feacalis* likely due to a large rectal stool burden that obstructed the bladder and the kidneys bilaterally as demonstrated by CT imaging. Collectively, findings from admission along with the patient’s history of chronic constipation quickly delineated diagnoses to a likely gastrointestinal etiology. Stagnation of fecal matter was likely at play and attempts at bowel cleanout were thereby quickly initiated. Differential diagnoses of chronic constipation leading to the buildup of large, stagnant, and hardened fecal mass, or a fecaloma, include functional causes like fecal retention, lack of fiber bulk in diet, and psychiatric conditions like depression or an eating disorder. Mechanical obstruction, as a broad category, is also on the differential and may be due to late presenting Hirschsprung disease, the presence of a pelvic mass, rectal stenosis, or postoperative stricture formation. Chronic constipation may be caused by pain on defection which may arise due to the presence of an anal fissure or foreign body, sexual abuse or perianal streptococcal infection.

Vasculitides may also present with gastrointestinal and renal symptoms as well as failure to thrive. These diagnoses, however, are systematic, often presenting with symptoms affecting more than 1 organ system due to the diffuse inflammation of blood vessels. For instance, while immunoglobulin A vasculitis may present with gastrointestinal symptoms ranging from acute, colicky abdominal pain to obstruction and subsequent perforation, the diagnosis also requires the patient to be younger than 20 years old, to have palpable purpura, acute abdominal pain, or granulocytes within the walls of small vessels.

An ovarian mass (eg, cystic teratoma), uterine fibroids, and lymph node swelling (reactive or malignant) are on the differential for a lower left quadrant tender mass.

In our patient, functional constipation due to limited oral intake was determined to be the leading diagnosis after interview and physical examination. Colonoscopy, ultimately performed to alleviate the stool burden, excluded both mechanical obstruction and causes of pain on defecation. Biopsies from the colon (sigmoid, descending and transverse), cecum, and ileum, demonstrated normal mucosa without significant histologic abnormalities. Mucosal biopsies from the upper gastrointestinal tract, including the esophagus, stomach, and duodenum were also found to be normal without histological abnormality. The entirety of the examined colon appeared enlarged during the diagnostic colonoscopy and an ulcer, without active bleeding, was visualized in the rectum. Collectively, histology and colonoscopy findings aided in ruling out inflammatory bowel disease.

Importantly, no significant increase in epithelial lymphocytes was identified on the duodenal biopsy. Biopsies from the middle, proximal, and distal esophagus were negative for increased intraepithelial eosinophils which aids in ruling out eosinophilic esophagitis. In addition, the lack of an inflammatory infiltrate in the mucosal biopsies and the lack of fibrinoid necrosis makes vasculitides less likely. No comment was made on the presence, or lack thereof, of eosinophils in the colon. Similarly, no comment was specifically made about the presence or absence of ganglion cells in the colon.

Pathology of rectal biopsies later ruled out a diagnosis of late onset Hirschsprung disease. Of note, the patient verbalized negative thoughts about self-image on initial presentation and a psychiatry consultation subsequently ruled out an eating disorder.

## Chronic Constipation

Chronic constipation is a common ailment in children and can often be classified as functional based on Rome IV criteria.^1^ Unfortunate complications can ensue if the condition is not promptly diagnosed and managed.^1^ One rare consequence of chronic constipation is stercoral colitis (SC), in which a fecaloma grows inside the bowel and eventually distends the colon to the point of compressing blood vessels and decreasing blood flow, causing ulceration of the bowel.^2^ SC has been described in the adult population, but it is rare in the pediatric population and has only presented secondary to psychiatric, developmental, or other conditions.

## Stercoral Colitis

Stercoral colitis is an inflammatory colitis that occurs due to colonic distension and fecaloma formation secondary to the prolonged impaction of fecal matter in the setting of chronic constipation. Fecalomas lead to the distension of the surrounding colonic walls and the compression of underlying vasculature which gives rise to pressure-induced necrosis and ulceration, typically in areas adjacent to the fecaloma. Without prompt intervention, areas of necrosis and ulceration may eventually perforate, a much-feared complication of SC that is associated with a mortality rate as high as 60%.^3^ Few documented cases of SC have been reported, and patients in which the condition has been described are typically elderly with a past medical history significant for chronic constipation,^4^ bed bound patients, particularly those with dementia^5^ or history of stroke,^6^ and patients with chronic opioid-use induced constipation.^7^ Even fewer cases of SC in younger patients have been reported and patients with the condition typically also present with comorbidities that include psychiatric conditions^3^ like anxiety and schizoaffective disorder, developmental disorders^8,9^ that include autism and cerebral palsy, or muscular dystrophies.^10^ This report presents the case of a 15-year-old patient, with a relatively brief history of chronic constipation, no other known or documented comorbidities on presentation who is not on any medications, presenting with AKI as the primary presentation of SC.

## Clinical Presentation

Stercoral colitis has a varied presentation that typically includes complaints of chronic constipation and abdominal pain. Chronic constipation and severe impaction may further present with urinary symptoms that include difficulty urinating, urinary retention, and dysuria secondary to a stagnation induced urinary tract infection. On physical examination, patients may have abdominal distension and tenderness, a palpable fecal mass in the lower abdominal quadrants and a palpable, hardened fecal mass in the rectal vault on digital rectal exam.

## Management

Treatment of SC includes aggressive attempts at a bowel cleanout with oral agents like senna, docusate, polyethylene glycol, or Dulcolax, as well as rectal enemas, such as the ones used for this patient, which included a combination of cottonseed oil, docusate, and normal saline. If needed, manual fecal disimpaction allows for the rapid alleviation of the stool burden as well as the reduction of the colonic intraluminal pressure and the associated risk of pressure-induced ulceration and perforation. In cases of SC complicated by perforation and subsequent peritonitis, surgical intervention is required.

## Pathology and Patient Course

For the patient being discussed, surgical pathology reports of the rectal biopsy taken during colonoscopy described an erosion with reactive changes but no evidence of dysplasia, consistent with the diagnosis of SC.

The patient recovered in the hospital and was discharged home with an extensive bowel regimen and adequate education to prevent continued constipation. The proteinuria and other renal abnormalities due to the AKI resolved without urologic sequelae and the bilateral hydronephrosis had resolved per follow up with pediatric urology at 2 and 12 months after admission. Labs prior to discharge demonstrate electrolytes entirely within normal limits, creatinine of 0.51 mg/dL (normal), BUN of 8 mg/dL (normal), albumin of 2.6 (persistently low), hemoglobin of 9.3 g/dL (low but improved), and a mean corpuscular volume of 81.7 fL (normal). Urine analysis was not repeated prior to discharge.

## Conclusion

As illustrated in this case report, AKI in an otherwise healthy pediatric patient, particularly in the context of chronic constipation and imaging suggestive of severe fecal impaction should provide sufficient evidence for the addition of SC onto the list of differential diagnoses. Including SC is crucial as its sequelae, which range from ischemic colitis and perforation to sepsis and multiorgan failure, can be circumvented with aggressive oral bowel cleanouts and potentially necessary manual fecal disimpaction. Investigating etiologies that give rise to SC and providing instructions on bowel rehabilitation is also critical for the outcomes of the pediatric patient.

## Author contributions

DKD: Contributed significantly to the collection, preparation, and analysis of this report. LA: Contributed significantly to the collection, preparation, and analysis of this report. MB: Contributed significantly to the collection, preparation, and analysis of this report.
